# Dance for health impact in a rural Scottish island community: understanding stakeholder experiences

**DOI:** 10.1093/heapro/daag069

**Published:** 2026-05-29

**Authors:** Emily Davis, Bethany Whiteside, Tiffany Stott, Lorna Paul

**Affiliations:** Department of Sport and Exercise Sciences, Durham University, Green Lane, Durham, DH1 3LA, United Kingdom; Institute for Medical Humanities, Durham University, Stockton Road, Durham, DH1 3LE, United Kingdom; Directorate of Research and Engagement, Royal Conservatoire of Scotland, 100 Renfrew St, Glasgow, G2 3DB, United Kingdom; Scottish Ballet, National Centre for Dance Health, 25 Albert Drive, Glasgow, G41 2PE, United Kingdom; School of Health and Life Sciences, Glasgow Caledonian University, Cowcaddens Road, Glasgow, G4 0BA, United Kingdom

**Keywords:** dance for health, health promotion, physical activity, creative health, multiple sclerosis, rural, community engagement

## Abstract

Research and practice around dance for promoting health and well-being are rapidly expanding, especially dance for chronic neurological conditions. Yet, research has primarily focused on assessing individual-level therapeutic outcomes, providing limited insight into broader stakeholder voices and community-level health impacts. In response, a longitudinal qualitative interview study was conducted to explore stakeholder perspectives on the community impact of a collaborative, community-centred dance for multiple sclerosis (MS) initiative in a rural Scottish island community. Repeated semi-structured interviews were undertaken with 19 participants, recruited purposively across three fieldwork visits. Participants included organizational partners, dance practitioners, musicians, volunteers, and dancers with MS. Interviews were transcribed verbatim and analysed using reflexive thematic analysis. Three themes were developed: (i) amplifying specialist community dance capacity, supporting individual practitioners and the island’s wider dance industry; (ii) promoting social capital and inclusion among the diverse local stakeholders involved, especially those living with MS; and (iii) providing a holistic well-being resource for participant dancers with MS, affording regular opportunities for physical, joyful, and meaningful activity through dance. By adopting a broader health promotion perspective, findings show that community-centred dance for health initiatives can generate a range of impacts in rural contexts both among and beyond the primary target participant group (i.e. people with MS), supporting capacity, inclusion, and well-being. Given the breadth of community impacts reported, findings also highlight the importance of maintaining such changes locally, suggesting the need for further consideration around organizational planning, support, and investment in the long-term sustainability of dance for health in rural contexts.

Contribution to Health Promotion

- Community-centred dance for health programmes tailored to specific chronic conditions can result in wider health promotion impacts both for and beyond target participant groups.
- Collaborative dance for health projects working across diverse stakeholders can foster linking, bridging, and bonding social capital.
- Supporting both organizational and individual capacity in delivering dance for health provisions is essential for their long-term sustainability in remote and rural contexts.

## Background

Dance engagement is a valuable resource for physical activity and creative health strategies to support health promotion across the lifespan (UK Chief Medical Officers 2019, All-Party Parliamentary Group on Arts, Health and Wellbeing and National Centre for Creative Health 2023). As an accessible and complex activity comprising physical, creative, emotional, and social components, dance supports various individual and population health outcomes, including the management of chronic conditions (Fong Yan *et al*. 2018, Sheppard and Broughton 2020). In the context of dance and health, dance for Parkinson’s disease (Parkinson’s) is particularly well-established in practice and research, evidencing the wide potential reach and benefits of dance for improving specific neurological symptoms and overall quality of life (Carapellotti *et al*. 2020, Senter *et al*. 2024). In response, dance provisions for other chronic neurological conditions are increasingly emerging, such as dance for multiple sclerosis (MS) (Sonke *et al*. 2021). With neurological conditions now recognized as the leading cause of health loss worldwide (Steinmetz *et al*. 2024), such health-promoting provisions are especially timely and consequential.

Dance provisions for health (dance for health) are offered across a range of contexts, from therapeutic interventions in healthcare settings to community projects in public spaces (Fortin 2018, Sheppard and Broughton 2020). Community-based dance for health initiatives often require engagement from diverse stakeholders, including dance practitioners, musicians, and community organizations (Fortin 2018). Despite this broad engagement, historically, dance for health research has narrowly focused on assessing individual therapeutic outcomes, with limited attention to understanding wider community participation and impacts (Sheppard and Broughton 2020, Petts and McGill 2024). As a result, certain dance for health experiences remain underexplored, potential benefits overlooked, and wider implications unknown (Dowlen *et al*. 2024). While a recognized dearth of studies has considered wider stakeholder voices, Dowlen *et al*. (2024) showed dance for health engagement can generate broader ‘ripple effects’, including promoting professional development and community networks among dance practitioners.

Despite this potential for broader impact, preliminary research around wider community experiences has also identified discrepancies and complexities in dance for health across different geographic contexts. In rural contexts, dance practitioners reportedly face challenges in accessing adequate dance for health resources, support, and networks, leading to experiences of ‘disconnection’ and ‘isolation’ (Collard-Stokes and Irons 2022). Owing to these access challenges in rural areas, participants can also experience barriers in accessing localized dance for health provisions, appropriately tailored to regional realities and priorities (Stickley *et al*. 2015, Herron *et al*. 2022). In turn, while access to dance provisions can yield their intended health impacts among participants, underresourced delivery can create an unintended well-being ‘irony’ (Collard-Stokes and Irons 2022) or paradox among more isolated rural dance practitioners. These challenges highlight the need to understand dance for health impact holistically, considering the experiences of both participants and delivery personnel in rural contexts.

Although such context-specific challenges are often emphasized in depictions of rural communities, their unique physical and social landscapes also present opportunities for particular social impacts from arts engagement (Balfour *et al*. 2018, Gibson and Gordon 2018). Rural communities are often characterized by high social capital (Scottish Government 2024c), encompassing trust, reciprocity, and networks (Putnam 2000). The contribution of social capital to the health of individuals and communities has been well-identified in research and policy (Putnam 2000, Szreter and Woolcock 2004, Kawachi and Berkman 2014); however, the relationship between social capital and creative health activities, such as dance for health, remains underexamined (Daykin *et al*. 2021, Ganga *et al*. 2025). Existing evidence suggests the potential of these arts activities to foster ‘bonding’ social capital among people with similar characteristics, but only limited research has considered their potential for building ‘bridging’ social capital between heterogeneous individuals and groups and ‘linking’ social capital across varying levels of institutional power and authority (Daykin *et al*. 2021).

Given the traditional individual-level therapeutic focus of dance for health research, these kinds of broader community-level health impacts have largely been neglected. Yet, these community-level impacts may be especially resonant in rural contexts owing to both their distinct strength and ‘shades’ (King *et al*. 2019) of social capital. Moreover, owing to resource constraints in rural areas, meaningful impact often extends beyond short-term therapeutic changes, relying on the continued contributions of community-based artists and stakeholders responsible for dance for health delivery (Menec *et al*. 2022). Still, understanding of dance provisions in rural settings remains limited, with existing research primarily focusing on remote and digital engagement (Herron *et al*. 2022, Bek *et al*. 2025) or performance- and output-focused participation (Anwar-McHenry *et al*. 2018). Consequently, little is known about stakeholder perspectives on the broader community impacts of locally developed and delivered dance for health provisions in rural contexts.

Specifically regarding dance for MS, as far as is known, no prior research has considered perceived impacts beyond individual-level therapeutic benefits to the primary target participant group, i.e. people with MS (Davis *et al*. 2023). Although a few dance for MS studies suggest benefits to social well-being (Piacentine *et al*. 2024), these insights remain limited to in-session support and in-group bonding, leaving wider community perspectives and impacts unexplored. Our study aimed to address these limitations in dance for MS research by exploring a range of stakeholder perspectives on the community-level impact of a collaboratively developed dance for MS project in the rural, remote Orkney Islands, Scotland. The project’s collaborative, community-centred design was well-suited to exploring the perspectives of both primary stakeholders (i.e. people living and dancing with MS) and secondary stakeholders (i.e. project dance practitioners, musicians, volunteers, and organizational partners).

Taken together, this study examined experiences and perceptions among these different stakeholder groups regarding the project’s community impact, guided by the research question: What is the perceived impact of the dance for MS project on involved local stakeholders and their community? By considering these stakeholder perspectives collectively rather than individually, the present study aims to extend understanding of dance for MS beyond individual therapeutic benefits to broader health promotion impacts in a rural island community.

## Methods

### Theoretical and contextual underpinnings

The Orkney Islands are an archipelago northeast of Scotland, comprising over 70 islands and islets (Dawson 2010). Orkney’s population is around 22 540, with over 17 000 people residing on the largest island, nearly half in the largest town and capital, Kirkwall (National Records Scotland 2022). Orkney reports the highest prevalence of MS globally, with any distinctively Orcadian risk factors remaining unclear and of ongoing research interest (Visser *et al*. 2012, Gay 2024). Consequently, MS and its associated symptoms present a particularly salient health challenge in Orkney and make localized MS support a high priority.

Orkney is a predominantly remote and rural area, as assessed by population density and accessibility criteria (Scottish Government 2024a). However, this study adopts a relational perspective from rural health research that extends understanding of remoteness and rurality beyond uni-dimensional spatial metrics, appreciating the dynamic interconnectedness of the various material, spatial, and social dimensions of rural places (Winterton *et al*. 2014). Aligned with this relational lens, the study is informed by health promotion frameworks that emphasize how health is shaped not only by individual dimensions and interventions, but rather in relation to wider community conditions and social forces (WHO 1986, Nutbeam 1998).

Consistent with this emphasis on social context, this study draws further on social capital theory, which highlights the role of social networks, relationships, reciprocity, and trust in health experiences and health promotion processes, including linking, bridging, and bonding forms of social capital (Putnam 2000, Szreter and Woolcock 2004, Kawachi and Berkman 2014). Correspondingly, the present study explores the socially constructed experiences and perspectives of stakeholders regarding the impact of the dance for MS project across the remote, rural Orcadian context.

### Project overview

Different from more typical community-based dance for health initiatives, the current project was funded and aimed at developing and delivering a community-centred model of dance for MS in Orkney (2021–23) (The Ideas Fund, no date; South *et al*. 2019). This involved mobilizing community assets, including through a collaborative partnership between two locally based organizational partners and two urban-based partners, the Royal Conservatoire of Scotland and Scottish Ballet (project lead). Scottish Ballet is a National Centre for Dance Health, with over a decade of experience in delivering dance programmes targeted to improving health and well-being, including their flagship neurological programmes (SB Health 2024).

Together, the project adapted Scottish Ballet’s existing dance programme model for MS, SB Elevate®, to meet the specific needs and priorities within the Orcadian context, including an emphasis on sustainable, community-led delivery and impact. This involved training local dance practitioners and musicians to deliver the dance for MS model via in-person and online training by Scottish Ballet staff, beginning in 2021. In July 2022, the first dance sessions launched at local dance studios. The project continued to expand over the project period, adding a second class offering and bringing on volunteers.

Dance sessions started with a meditation, progressed through seated lower- and upper-body exercises and standing full-body activities (seated and supported options provided), and concluded with a social café, social time with refreshments. Dance movements ranged from structured, practitioner-led sequences to improvisational, dancer-led creative tasks, drawing inspiration from various dance styles and different music genres, particularly Scottish traditional music.

Each session lasted approximately 90 minutes and was led by one dance practitioner and one support practitioner, accompanied by live music, and supported by volunteers. Sessions were free of charge for people with MS and their companions, and community artists received the same remuneration as other Scottish Ballet artists. Approximately 10–15 individuals attended sessions, including 5–8 dancers with MS, 2 dance practitioners, 1 musician, and about 2 volunteers and 2 companions.

### Study design

A longitudinal qualitative design was utilized to enable understanding of the project’s developing impact over time rather than providing a ‘static snapshot’ (Weiler and Grant-Smith 2024) as in prior dance for MS research (Davis *et al*. 2023). Our study was guided by an interpretivist paradigm centring stakeholder perspectives as both socially constructed and contextually situated (Lincoln *et al*. 2011). Serial or repeated interviews were used to understand these developing stakeholder perspectives. Conceptually, repeated interviews suited the current study, with multiple interviews recommended when capturing change over time and considering complex topics, aspects central to understanding the ongoing impact of dance in a unique island setting (Murray *et al*. 2009, Read 2018). Practically, serial interviews also suited the study, helping build trust over time and accommodate the schedules of key community stakeholders (Read 2018). The study was informed by Braun and Clarke’s (2025) values-based guidelines for reporting qualitative research. Ethical approval for the study was granted by the Royal Conservatoire of Scotland in July 2022 (reference number: EC2152). This study forms part of the lead author’s doctoral research, from which the data reported were derived (Davis 2025).

### Study sample

Participants were recruited by E.D. during in-person visits to Orkney by the Glasgow-based team members, E.D., B.W., and T.S., at three key project time points: Phase 1: first session launch (July 2022), Phase 2: project mid-point (November 2022), and Phase 3: project funding endpoint (March 2023). E.D. invited individuals to participate at local events, such as dance sessions, providing details on the study’s purpose of better understanding the project and its impact. This sampling approach was purposive, targeting information-rich stakeholders across levels of project involvement, while also shaped by convenience in who was available during visits (Robinson 2014). E.D. is an American female researcher with expertise in qualitative research and dance for health, but no prior connection to Orkney. Thereby, E.D. was an ‘insider’ in relation to the topic of study and ‘outsider’ in the island context (Braun and Clarke 2025).

Nineteen participants took part in the study, including stakeholders devising, delivering, and participating in the dance project (Table 1). All stakeholders approached participated in at least one interview. Participants’ study involvement often mirrored their project involvement timeline, such as volunteers joining in Phase 3. Some participants declined follow-up interviews due to scheduling conflicts or personal circumstances. Sample size and the number of interviews undertaken across project phases were informed by considerations of information power given the specificity of the involved stakeholder groups (Malterud *et al*. 2016). All participants provided written, voluntary informed consent before participation, with consent forms detailing limitations in the anonymization possible due to the public nature of the project and the small nature of the community context. Still, only limited participant details are provided to maintain anonymity. Role descriptors and phases (see Table 1) are used to reference participants and denote their viewpoints.

**Table 1:** Interview participants.

Participants	Phase 1 (P1)	Phase 2 (P2)	Phase 3 (P3)
Scottish Ballet Partner 1 [SB1]	X	X	X
Scottish Ballet Partner 2 [SB2]	–	–	X
Community Partner 1 [CP1]	X	X	–
Community Partner 2 [CP2]	X	X	X
Community Research Partner [CR1]	X	X	X
Dance Practitioner 1 [DP1]	X	X	X
Dance Practitioner 2 [DP2]	X	X	X
Dance Practitioner 3 [DP3]	X	X	–
Dance Practitioner 4 [DP4]	–	X	X
Dance Practitioner 5 [DP5]	–	X	–
Musician 1 [M1]	–	X	X
Musician 2 [M2]	–	X	–
Musician 3 [M3]	–	X	–
Volunteer 1 [V1]	–	–	X
Volunteer 2 [V2]	–	–	X
Dancer 1 [D1]	X	X	X
Dancer 2 [D2]	–	X	X
Dancer 3 [D3]	–	X	X
Dancer 4 [D4]	–	–	X

### Data collection

E.D. conducted 37 interviews total across the three time points, between July 2022 and March 2023. Interviews were conducted in a flexible mode depending on each individual’s preferences, including the setting (e.g. coffee shops, participants’ homes, online), format (one-to-one, pairs, small groups), and length (25–150 minutes), with nearly all interviews undertaken one-to-one, in-person (*n* = 32). A semi-structured interview guide was employed, with a focus on the perceived individual and community impacts of the dance project (Supplementary Table S1). Semi-structured interviews suited the present study by centring discussion on topics related to impact, while also allowing flexibility to probe emergent topics appropriate to each participant’s contextual location (Ritchie *et al*. 2014). All interviews were audio-recorded following participant permissions and transcribed verbatim with assistance from NoScribe (Dröge 2024), AI-powered transcription software. E.D. prepared each interview individually by actively listening to each recording while manually reviewing each transcript before importing all transcripts into NVivo (Version 14) software (Lumivero 2023) for analysis.

### Data analysis

E.D. conducted reflexive thematic analysis, following Braun and Clarke’s six-phased approach (2021). Phase (1), data familiarization, occurred immediately after each data collection phase by repeatedly reading and listening to interviews, alongside reflective note-taking to identify particularly salient concepts, e.g. ‘new social connections’. Phase (2), coding, was conducted inductively for each transcript, using both semantic and latent codes. Coding was data-driven, for example, utilizing participants’ own terms like ‘jack-of-all-trades’ as code labels. Phase (3), generating initial themes, was started separately for each data collection phase, involving active, iterative clustering of codes to explore shared meanings, such as grouping well-being-related codes like ‘experiencing joy’.

Completing Phase (3) and commencing Phases (4), (5), and (6), developing, refining, and writing-up themes, occurred once the entire dataset was coded. Consequently, the iterative process of theme construction was informed by the repeated nature of visits and interviews, allowing themes to be further contextualized and reviewed against the full dataset, including reflecting both changes and constants over time. For example, the theme generated (3) around capacity-building evolved from an initial focus on immediate impact to broader, ongoing implications owing to increased local capacity. Throughout theme development (4) and refinement (5), reflexivity was actively practiced, for example, critically negotiating divergent perspectives on perceived social impact to develop a more nuanced theme around social capital.

B.W. and L.P. provided feedback and opportunities for further critical discussion and reflexive thinking on developing and refining the analysis. Our ongoing dialogue benefitted from our different experiences across dance, health, and qualitative research, enabling informed, critical consideration of the more dance-based aspects of impact relative to health determinants. Likewise, each team member’s varying exposure to the dance project and the Orcadian context helped balance insider insights and critical distance during analysis. Overall, these continuous, collective, and reflexive processes, alongside the audit trail of documentation, including thick description and verbatim quotes in the reporting, maintained trustworthiness in our analysis (Ritchie *et al*. 2014).

## Results

Three main themes were identified concerning the community impact of engagement with the dance for MS project: (i) amplifying specialist community dance capacity, (ii) promoting social capital and inclusion, and (iii) providing a holistic well-being resource.

### Amplifying specialist community dance capacity

Orkney enjoys a rich arts and cultural landscape, including a long history of ‘world-class’ [CP1] Scottish traditional music and a diversity of dance offerings. This provided a strong foundation of expertise and enthusiasm to build specialist capacity in creative health, specifically, dance for health. For most involved community artists (i.e. dance practitioners and musicians), the project inspired and supported a new specialist skillset in dance for MS delivery.

I’d heard of the dance health background, but never really seen it in action. And obviously, when Scottish Ballet came up we were all very inspired. It was really just like ‘Oh, this is really great.’ And it just kind of opened my eyes to that and thinking yeah, Orkney really does need this. (Dance Practitioner 1, P1)

This upskilling in dance for MS was particularly welcomed locally, not only due to community artists’ interest in creative health, but also the local relevance of MS support, whereby ‘everyone knows someone who’s affected by MS’ [CP2]. Practically, the remoteness characteristic of island living also often limited the availability of advanced training locally and entailed high expense for training externally, making the community-based training particularly appropriate and valuable for local artists.

Because we can’t just get a train to another town; we are where we are. There isn’t the option to go somewhere else for a day to see, or even just on the training aspect, if I wanted to get trained in dance for MS. (Dance Practitioner 2, P1)

Most local dance practitioners and musicians reported benefits from both the formal Scottish Ballet-led training and learning ‘on-the-job’ [DP1], helping expand their creative ‘repertoire’ [M1]. For dance practitioners, teaching dance for MS required creativity beyond their usual practice, fostering new capabilities and confidence transferable to their other classes. Similarly, musicians developed their skillset over time, experimenting with different ‘tunes’ [M2] and instruments to best suit dance for health delivery.

Never really been in a position to interact with people like the programme enables you to do. It’s completely different. And it’s like I say, tapping into the creative side of the brain that we’ve not had to use because it’s pre-set choreography or syllabus. (Dance Practitioner 3, P2)

Beyond individual practitioner development, the project inspired broader developments in capacity and engagement locally over time. A few dance practitioners were motivated to further upskill in dance for Parkinson’s delivery, and several participant dancers with MS and volunteers enrolled in other local dance classes. The project also reportedly increased awareness around dance and its relationship to health across the Orcadian community, helping amplify the distinct role and value of the local dance industry.

If we can bring up that value for like all the different types of dance, not just your childhood, doing it for a couple of years – but to get better balance or coordination. I think it’s important and it will help everybody in the dance industry here to bring that value up. (Dance Practitioner 2, P3)

The project also directly supported the local dance industry in the short term, ‘generating jobs, generating opportunities, and generating money for Orkney’ [CP2]. In turn, several community artists expressed the economic value of increased income and expanded professional portfolios via the project, contributing to feeling ‘valued’ [M1] in their creative profession.

As a musician, you feel valued. And like so many people in the world think that musicians just play for the love of it, and nothing else. It’s like, well no it’s a job, and it’s a skill, and it takes a lot of work to get to where you are. So, you should be reasonably paid. (Musician 1, P2)

Supporting the local dance community, particularly the specialist expertise of community artists, was viewed by all stakeholder groups as important to ensuring the ‘longevity’ [DP2] of dance for MS delivery and impact, allowing expertise to remain in the community even once the project’s funding concluded. Yet, some project partners also raised concerns around the limited organizational capacity developed locally during the project, with Scottish Ballet maintaining the central organizational role. In turn, while local specialist delivery capacity was strengthened among community artists, questions remained around general organizational capacity for leading the future of dance for MS in Orkney.

Who kind of organises everything? Because it will need an organiser. We can’t just say, you know, just organise it between yourself, just someone will have to take a lead. (Community Partner 2, P3)

### Promoting social capital and inclusion

By design, the project connected diverse individuals, groups, and organizations around the ‘common goal’ [CP2] of supporting Orkney’s MS community through dance. Early on, this entailed establishing the cross-sector, multi-site partnership between locally based community organizational partners and external, urban-based organizational partners. Through linking local and national organizations, project partners viewed this collaboration as a means of increasing access to institutional capacity and capital, namely of the project’s lead, Scottish Ballet, alongside encouraging mutual learning across institutional levels, sectors, and geographic locations.

I think it’s really important to reach different communities that perhaps wouldn’t usually be connected with something like Scottish Ballet […] We learn from the community, and the community can also benefit from all that Scottish Ballet has got to offer as well. (Scottish Ballet Partner 2, P3)

Regarding this mutual learning, many stakeholders, across project partners, delivery artists, and participating dancers, particularly noted the key role of community members in this collaborative partnership. Involved community members helped promote local engagement, for example, with community artists drawing on local knowledge and networks to tailor provisions to the island community, while still capitalizing on this ‘link’ with Scottish Ballet.

[Elevate] will be kind of slightly different to where the location is from cities, to obviously, we’re up in the North of Scotland […] we’ve kind of created our own sort of idea on it [Elevate…] but still keeping linked with Scottish Ballet, and I think that’s really important. (Dance Practitioner 1, P3)

The dance project also provided an opportunity for bridging different individuals and social groups within the island community, including across social differences like generations, professions, and illness experiences. Similar to other remote, ‘small’ [CP2] communities, Orkney’s social fabric was characterized by social closeness and interconnectedness, alongside the social isolation and distance inherent in ‘living on an island’ [D1]. Consequently, some community stakeholders shared overlapping social histories and networks, but the project mostly connected different individuals and groups for the first time, including some community artists previously working ‘in silos’ [SB1] or ‘separate worlds’ [M2]. For example, for most of these local artists, this new bridging social capital provided benefits, such as increased peer support, reduced professional isolation, and new collaborative opportunities.

So it’s nice to have someone else because it’s a very lonely job like teaching, so it’s nice to have someone else [they] can like bounce ideas off and share experiences with. (Dance Practitioner 3, P1)

The dance sessions themselves were described as socially ‘inclusive’ [V1], ‘welcoming’ [D2], and ‘homely’ [DP1], providing a ‘safe space’ [D1] that promoted bridging processes across the various groups involved, from local artists to locals with MS. Moreover, most stakeholders across project positions expressed feeling they held an active role in the sessions and group, rather than, for example, musicians being ‘handed some sheet music, saying, “Monkey, go play this.”’ [M1]. In turn, sessions were characterized as ‘an equal playing field’ [SB2], where individuals felt involved and included, whether in paid or unpaid roles or living with or without MS.

There’s no differentiation really between who’s a musician, a dancer, a volunteer, or a person with MS. It’s a very inclusive class. And there’s no hierarchy in it. Everybody’s accepted and just does their own little bits that’s needed. (Volunteer 1, P3)

Many stakeholders from delivery artists to dancers with MS referenced the enjoyment and value of ‘mixing’ [D2] on equal footing with people from different backgrounds and ‘all walks of life’ [M1] in their community, with some noting the ‘social side’ [CP1] as ‘just as important as the dance’ [D1]. The social café was frequently referenced as key in providing dedicated time and space for individuals to grow closer on a ‘more connected level and more personal level’ [DP4].

I think the social café at the end, it’s just priceless. That’s really, that’s part of how we have time to actually ask, ‘Oh, do you have kids?’ And asking all those questions. (Dance Practitioner 2, P2)

As relationships deepened across social groups in sessions, over time, awareness and mutual understanding developed among involved community members, particularly around more ‘hidden’ [M2] community voices. Project engagement reportedly ‘opened the eyes’ [M3] of several community artists to the lived experience of MS, understanding ‘not all disabilities are visible’ [M1]. Also, participant dancers were perceived to develop greater appreciation for the skills and contributions of local artists, holding implications for social inclusion outwith the project itself.

It’s opened their eyes to being like this is what a musician could look like, or we’re not all the same, and there’s actually lots of us here in Orkney. And they maybe don’t see us? (Musician 3, P2)

Specifically, for some people with MS, participation in weekly sessions also mitigated social isolation and expanded social support. The project generated some social ‘ripples’ [CR] for certain community members’ social networks and social participation; for example, some dancers with MS engaged more frequently with other community resources and now greeted other stakeholders during chance local encounters.

[MS] It’s quite isolating. No, I take that back. It’s very isolating. And I just like the thought that my window of opportunity of speaking to people has opened up. (Dancer 1, P2)

Over the course of the project, dancers with MS, volunteers, and community artists also reported social bonding through a shared purpose and collective identity as part of ‘something bigger’ [SB1] within their community. Several of these stakeholders expressed pride in being part of the dance project, group, and collective ‘we’, with a few dance practitioners also feeling proud to be associated with Scottish Ballet.

I love being a part of this group. Not just being a part of Scottish Ballet, but being a part of a group that we’re able to help these people that, you know, this is something they’ve never had before in Orkney. So yeah, I think it’s a very proud moment for us all to be a part of that. (Dance Practitioner 1, P3)

By the end of the project period, social connections were described by those in sessions (i.e. community artists, volunteers, and dancers with MS) as particularly strong, close, and integrated, becoming ‘like a family’ [DP2] and ‘wee community’ [DP1] over time by building ‘trust’ [D2] and ‘affection’ [D1] between one another. Overall, the inclusive and supportive dynamics of the dance sessions helped foster a dance for MS community locally, whereby each individual’s presence was noticed and valued.

[Dancer] said they missed us when we weren’t in class. They said ‘we missed you.’ (Dancer 3, P3)

### Providing a holistic well-being resource

Especially for the primary stakeholders, people with MS, the dance project provided a holistic well-being resource. Weekly sessions provided a needed local opportunity for accessible and affordable physical activity for the MS community, which was described as ‘fun’ [D4], ‘joyful’ [V1], and ‘different’ [D3] compared to other exercise offerings like gym classes, due to the music, creativity, and social interaction. Together, these different dimensions encouraged positive emotions among those with MS, along with some other involved stakeholders, like older adult volunteers.

The music just carries you along. I love it, I love it, I love it. (Dancer 1, P3)

I was quite glad that I’m not working in a charity shop regularly anyway. I don’t mind doing it now and again, but it certainly wasn’t as joyful as an Elevate class. (Volunteer 1, P3)

As a physical activity, most dancers with MS noted improvements in their physical well-being from dance. Oftentimes, dancers connected these perceived physical improvements in motor functions to maintaining important activities in their ‘everyday’ [D2] routines, such as keeping their driving licence. A few participant dancers also reportedly practiced movement exercises from sessions at home, offering creative strategies that further supported dancers’ mobility and MS management day-to-day.

Some have been telling us how they do it [relevés] at home holding on to the kitchen sink. They’re kind of using what they learn in class and putting it into their daily lives. (Dance Practitioner 4, P3)

Improvements in mental well-being were also noted across the different stakeholder groups following dance sessions, with one volunteer observing, ‘everybody comes out of the class happier than they went in’ [V2]. Particularly for dancers with MS, along with feeling ‘buoyed up’ [D1] after sessions, some described wider contributions to mental well-being, particularly in using the ‘invigoration’ [V1] and ‘energy’ [D1] from sessions to support ‘getting out more’ [D3] in their community and help boost the mental well-being of others around them in their daily lives.

I think it’s very good for your mental well-being. It’s hard if you get in a rut sort of thing, ye ken. Yeah, I think you know it’s good to get out and join in things. (Dancer 4, P3)

Another key way dance supported the mental well-being of dancers with MS was by providing a sense of purpose. For some dancers, sessions provided ‘a reason’ [D1] and ‘something to look forward to’ [D1], with a few organizing their schedules and lives around weekly attendance.

I’d say if we weren’t going, we’d be sitting at home, bored. Because you have to, wherever you’re living, you need to communicate with other people. (Dancer 3, P3)

Likewise, other stakeholders involved in session delivery also described feeling a sense of purpose. For dance practitioners, several expressed benefiting from seeing the meaningful impact of their delivery on participating dancers and, in turn, understanding the purpose and value of their teaching. Also, across both community artists and volunteers, especially volunteers retired from work, several shared finding purpose in helping their local MS community, describing mutual ‘rewards’ [DP1] for both participating dancers and those facilitating sessions.

It’s just that they’re so eager, they’re so involved and they’re so thankful as well, which is really humbling for me. Like each week they always thank us for a lovely class and how it makes them feel. (Dance Practitioner 4, P3)

I enjoy meeting the people, trying to help them, and getting to know them more each week. (Volunteer 2, P3)

Especially as the dance sessions became meaningful and ‘embedded’ [SB2] in community stakeholders’ lives, their continuation was considered particularly significant. Near the end of the project’s funded period, several participant dancers expressed ‘devastation’ [D1] at the possibility of sessions being discontinued. In turn, across stakeholder groups, there was a shared sentiment around planning and ensuring the project’s community impact was sustained.

The amount of effort people make, and how they’ve really embedded it as part of their life, their weekly routine […] It shows the importance is huge. And so if it was to go, and it wasn’t to be sustained, then that’s huge and massive. (Scottish Ballet Partner 2, P3)

## Discussion

The findings of the present study demonstrate the community impact of engagement with a dance for MS project within a remote, rural island community, supporting local capacity, social capital, and holistic well-being. These findings expand on a growing evidence base supporting the benefits of dance provisions for neurological conditions, centring on dance for MS. Compared to previous dance for MS research (Davis *et al*. 2023), this study extends the dominant focus on individual-level therapeutic effects to encompass broader stakeholder perspectives on community-level health impacts. As the first study to assess dance for MS from a broader health promotion lens within a rural island setting, the study offers new insights into the potential of dance for health as a creative approach to address pressing public health challenges in remote and rural contexts, such as increasing prevalence of chronic conditions and limited access to specialists and specialist health training (Heaton 2022, NHS Orkney 2024).

### Addressing specialization versus organization

The current dance for MS project addressed noted rural access restrictions to specialists and specialist health training by increasing local access for community artists to specialist dance for health training and thereby widening access for community members with MS to specialist health-promoting provisions. Given the noted lack of accessible professional development opportunities for rural UK dance practitioners (Collard-Stokes and Irons 2022), this locally offered upskilling was consequential to not only building project delivery capacity but also enhancing rural artists’ professional skills, portfolios, and entrepreneurship (Duxbury 2021). Moreover, for the local MS community, amid continually diminishing rural services following COVID-19 (Bryce and Johnson 2021, Fixsen *et al*. 2021), these localized developments were key to addressing gaps in specialist MS provisions. Across stakeholder groups, the project also encouraged further dance engagement, whether in further upskilling by artists or local dance offerings by volunteers, demonstrating the potential for ‘ripple effects’ (Dowlen *et al*. 2024) in dance and health beyond the project’s immediate scope and target group, i.e. health promotion for MS. Compared to remotely or digitally delivered dance in rural communities (Hill *et al*. 2021, Herron *et al*. 2022), findings demonstrate the unique potential of locally developing capacity to foster wider community ripple effects in the regional dance sector.

Yet, within rural contexts, sustaining capacity presents particular challenges, in part due to population and infrastructure constraints (Alexander 2016, Scottish Government 2024b). In turn, although specialist practitioner capacity was strengthened, building general organizational capacity to continue the project beyond its funded endpoint was a challenge. In capacity-building health promotion interventions, the World Health Organization (WHO) explains that the advancement of knowledge and skills among practitioners alone is insufficient, requiring also ‘the expansion of support and infrastructure for health promotion in organizations’ (WHO 2021). Aligning with this WHO perspective, other rural dance initiatives have noted the significance of sufficient organizational capacity, with Menec *et al*. (2022) articulating the key role of voluntary support in filling structural ‘resource gaps’. That said, relying on this kind of collective, voluntary support raises concerns around individuals in rural communities assuming responsibilities without sufficient perceived or material structural support (Collard-Stokes and Irons 2022). Consequently, while our findings suggest locally developing dance for health initiatives can enhance ‘specialization’ among local artists, further consideration is needed on how to establish the ‘organization’ responsible for implementation to support sustainable community delivery and impact in rural areas.

### Beyond social bonds, towards social bridges

While research concerning participatory arts mostly cites positive impacts on bonding social capital among participants with shared characteristics (Daykin *et al*. 2021), the present findings show the potential for broader impacts on bridging and linking social capital. Regarding linking social capital, Scottish Ballet secured the project funding and contributed their resources, vertically linking local community organizations and stakeholders with their wider institutional support structures and status (Szreter and Woolcock 2004). Reciprocally, the expertise of local ‘insiders’ was essential to the project’s relevance and engagement locally. These findings offer new insights in the context of social capital and participatory arts projects, showing the mutually beneficial potential of developing ties between outside resources and insider expertise through collaborative partnerships, especially in rural settings with more limited organizational infrastructure. In addition, by focusing on a rural community with rich cultural resourcing, this study builds on an emerging international dialogue around the value of the ‘rural creative class’ (Duxbury 2021), illustrating the contributions and capital of locally based stakeholders from an asset rather than deficit-based lens in combined internal and external partnerships in rural contexts. By considering these linkages across contexts, findings illustrate the wider social connections and implications of multi-site and multi-organization projects, providing insight into social impacts beyond social bonding among participants.

Alongside linking social capital, a widely noted community impact was on bridging social capital, horizontally bridging diverse groups and individuals within the island community (Putnam 2000, Kawachi and Berkman 2014). The dance project reportedly built new connections across local dance practitioners, musicians, volunteers, and people with MS, with implications for supporting cross-sector professional collaborations, mitigating social isolation, promoting social inclusion, and widening social participation for people with MS. Although research on bridging capital is relatively limited in arts and health (Daykin *et al*. 2021), similar bridging potential was reported from participatory singing among Aboriginal and Torres Strait Islander Australians, including those with chronic conditions at risk of social isolation (Sun and Buys 2013). While acknowledging the distinct cultural and historical contexts of Indigenous Australian and UK island communities, our findings show that dance, like singing, can support bridging connections across social differences, including by promoting new social interactions, relationships, and sources of community participation (Sun and Buys 2013). Together, these findings invite further consideration of the potential of different artistic, physical, and social activities engaging diverse social groups in a given community, such as participatory music or walking groups, to not only strengthen social bonds but also build new social bridges.

### From well-being for multiple sclerosis to rural community well-being

The social dimensions of the dance for MS project were central to the holistic well-being experiences of the primary stakeholders, people with MS, for whom social capital is particularly challenging to maintain yet key to both health outcomes and health promotion processes (Reyes *et al*. 2020). Alongside enhancing social well-being, those with MS also described the physical and creative aspects of dance as influential to changes in physical and emotional well-being. Over time, sessions also supported emotional well-being not only by providing a sense of purpose for some dancers’ weekly lives, but also for community artists and volunteers in finding purpose in fulfilling a valuable community service. Especially as the prevalence of chronic conditions continues rising in rural communities (NHS Orkney 2024), such opportunities for meaningful engagement are particularly important for health promotion among those living with conditions like MS (Mozo-Dutton *et al*. 2012). Moreover, with increasingly ageing populations in rural areas, these benefits of meaningful engagement could also extend to older adults volunteering in such initiatives (Farmer *et al*. 2011, Pettigrew *et al*. 2020), as denoted among volunteers in dance for dementia provisions in rural Canada (Herron *et al*. 2022). Our findings echo this potential in the context of dance for MS in a rural Scottish island community, with these kinds of voluntary benefits largely overlooked in dance for health. Together, these findings shed light on the unique potential of dance for health to provide well-being benefits more broadly, both for participating dancers and other involved community members.

Furthermore, in part owing to the project’s community-centred design, well-being shifted from a target outcome only for participants with MS to a collective experience among all involved community members. For most community stakeholders, the project became a source of collective well-being, purpose, and identity, promoting social bonding by offering a sense of belonging to something bigger within their community. These community impacts align with other participatory arts initiatives (Daykin *et al*. 2021), including dance provisions in rural, remote Western Australia (Anwar-McHenry *et al*. 2018). Still, these collective impacts have generally been neglected in studies of disease-specific dance interventions, which are often contrasted with more general community health promotion initiatives in dance for health (Sheppard and Broughton 2020). However, current findings demonstrate the benefits of considering well-being collectively and health promotion holistically, looking across the continuum of individuals devising, delivering, and participating in dance for health programmes. This wider community health promotion lens opens up consideration of different forms of impact, appreciating the benefits of dance beyond a specific disease, while also recognizing that a specific disease in a specific context may present a particularly galvanizing point for regional support and community bonding, such as MS in Orkney. That said, even with this strong community support, concerns were raised about sustaining project impact without coordinated support and funding from a centralized external organization. Consequently, while the current dance for MS project resulted in a range of community benefits during its funded period, such initiatives must also account for their continuation or the consequences of their completion locally when engaging with remote, rural communities.

### Strengths and limitations

This study is among the first qualitative studies of dance for MS and among the few dances for health studies to focus on a rural context or to consider diverse stakeholder voices. The study’s strengths lie in its novel focus on MS and a rural island context and its inclusion and synthesis of the diverse voices involved in dance for health. That said, since the sample was limited to individuals closely involved with the project, this limited representation from other community members, such as less engaged or disengaged participant dancers, whose perspectives might have offered alternative insights on community impact.

The study’s longitudinal design enabled more in-depth understanding of dance for MS impact through repeated engagement with participants and their perspectives. This longitudinal approach enhanced credibility, enabling the verification of accounts over time and critical reflection on evolving experiences. Still, the study remained context-specific and time-specific, limiting transferability to other communities and provisions and limiting understanding beyond the project’s funded period. As this was a qualitative study, findings are not intended to be generalizable, but the detailed project and contextual descriptions could help determine their transferability to similar settings.

Reflexivity was actively practiced throughout, attending to our underlying assumptions about dance and health, particularly in the distinct island setting. While this reflexive, iterative process among team members enhanced credibility, greater involvement of community voices during analysis may have provided further ‘insider’ insights and further enriched interpretations. Nonetheless, continued reflexivity and engagement in the field and with community members underpin the trustworthiness of the present study.

### Future research

Future work should expand on this study’s strengths by further exploring broader community perspectives and broader potential ripple effects of dance for health. Further adoption of longitudinal study designs could help capture these broader impacts and more comprehensively understand the long-term implications of such projects. Future research should also address limitations in the present study, considering challenges around non-participation, structural support, and long-term sustainability. There is also scope to extend such inquiry beyond dance, considering other leisure, physical, and creative activities.

## Conclusion

The present study provides qualitative evidence of the potential for a community-centred, collaborative dance for MS project between national and local partners in a rural island setting to promote community impacts, including amplifying local capacity, promoting social capital and inclusion, and enhancing holistic well-being. These diverse impacts were reflected across a diverse range of stakeholder perspectives, although concerns remained around sustaining the project’s delivery and impact locally beyond its funded period. Future research and practice should further consider means of promoting long-term, community-led dance for health initiatives in rural and remote contexts that support and strengthen local leadership and infrastructure.

## Supplementary Material

daag069_Supplementary_Data

## Data Availability

The full qualitative dataset generated and analysed during this study is not publicly available due to a residual risk of disclosure in the data. Please contact the corresponding author about obtaining access to the data.
