# Supplementary material for: Dance for health impact in a rural Scottish island community: understanding stakeholder experiences
Source: Health Promot Int. 2026 May 29;41(3):daag069. doi: 10.1093/heapro/daag069 (PMC13221699; doi:10.1093/heapro/daag069)
Supplement: daag069_Supplementary_Data [file daag069_supplementary_data.docx]

# **Supplementary Table 1**

# Topic Guide for Interviews with Stakeholders

| **Domain** | **Sample Topics** |
| --- | --- |
| **Background** | - Demographic background - Project role - Motivations for project engagement - Past experiences with the arts, health, or arts and health - Understanding of MS context in Orkney - Perceptions of dance for health in Orkney |
| **Implementation** | - Role and experience with project implementation over time - Experience with project collaboration and teamworking - Experiences/perceptions of project implementation - Perceptions of project reality vs expectations - Barriers and facilitators to project implementation - Considerations of learning over time - Hope for the project future |
| **Impact** | - Experiences/perceptions of personal impact - Experiences/perceptions of health and well-being impact - Experiences/perceptions of social impact - Experiences/perceptions of changes to daily life - Experiences/perceptions of professional impact - Experiences/perceptions of wider community impact - Understandings of changes in impact over time |
